# Change Management and Digital Innovations in Hospitals of Five European Countries

**DOI:** 10.3390/healthcare9111508

**Published:** 2021-11-05

**Authors:** Petra Hospodková, Jana Berežná, Miroslav Barták, Vladimír Rogalewicz, Lucie Severová, Roman Svoboda

**Affiliations:** 1 Department of Economic Theories, Faculty of Economics and Management, Czech University of Life Sciences Prague, Kamýcká 129, 165 00 Prague, Czech Republic; hospopet@fbmi.cvut.cz (P.H.); severova@pef.czu.cz (L.S.); 2 Department of Biomedical Technology, Czech Technical University in Prague, 272 01 Kladno, Czech Republic; berezja1@fbmi.cvut.cz (J.B.); rogalewicz@fbmi.cvut.cz (V.R.); 3 Department of Master Study Programs, Faculty of Health Studies, J. E. Purkyne University in Ústí nad Labem, 400 96 Ústí nad Labem, Czech Republic; miroslav.bartak@ujep.cz

**Keywords:** change management, healthcare, digital innovations

## Abstract

The objective of the paper is to evaluate the quality of systemic change management (CHM) and readiness for change in five Central European countries. The secondary goal is to identify trends and upcoming changes in the field of digital innovations in healthcare. The results show that all compared countries (regardless of their historical context) deal with similar CHM challenges with a rather similar degree of success. A questionnaire distributed to hospitals clearly showed that there is still considerable room for improvement in terms of the use of specific CHM tools. A review focused on digital innovations based on the PRISMA statement showed that there are five main directions, namely, data collection and integration, telemedicine, artificial intelligence, electronic medical records, and M-Health. In the hospital environment, there are considerable reservations in applying change management principles, as well as the absence of a systemic approach. The main factors that must be monitored for a successful and sustainable CHM include a clearly defined and widely communicated vision, early engagement of all stakeholders, precisely set rules, adaptation to the local context and culture, provision of a technical base, and a step-by-step implementation with strong feedback.

## 1. Introduction

There is no doubt that healthcare facilities are changing constantly, and these changes are challenging for all stakeholders [1]. Change management has been defined as the process of continually renewing an organization’s direction, structure, and capabilities to serve the ever-changing needs of external and internal customers. Change management is influenced by national context and may vary between different countries [2].

Burnes [3] says that up to 60% of all change projects, not just those in the healthcare context, fail due to poor change organization. One must also keep in mind that changes come in many different forms and on different scales with some changes being only minor or incremental, while others are more profound. This makes evaluation of the relative success or failure of a change rather difficult. Considering the speed with which changes are introduced to the healthcare sector, organizations must be ready to anticipate, implement, and manage them [4]. Technologies as well as political and social pressures guarantee that the healthcare provided by medical professionals will continue changing and evolving over time. Embedding CHM and change leadership firmly in an organization’s culture will make that organization more prepared for any future change [5].

The topic of change management can be studied from different perspectives, above all from the healthcare system perspective and from the healthcare provider (hospital) perspective. While the former approach has been quite frequently applied and there exist a number of professional publications as well as internationally recognized guidelines in this area (e.g., [6,7]), the latter one is less described and the results less implemented. Whatever perspective we speak about, the change should always go in line with the so-called Quadruple Aim, i.e., enhancing patient experience, improving population health, reducing costs, and improving the work life of health care providers including clinicians and staff [8].

Current research shows that change management poses different requirements on different management levels. While the top management supports and highlights the importance of a change by raising awareness and providing resources, middle management plays a key role in the implementation of the change. Relationships between top and middle management are also of crucial importance as it has been shown that the behavior of top management affects middle management engagement intensity [9]. Unfortunately, according to Birken et al. [10], middle management often receives very little support from top management.

The objective of this study is to evaluate the quality of systemic CHM and readiness for change in five Central European countries. The secondary goal (based on the results of the analysis) is to identify trends and upcoming changes in the field of digital innovations in healthcare. More detailed examination of this issue was also supported by numerous synoptic reports predicting such developments, see e.g., [11,12,13,14]. We will highlight potential shortcomings and the lack of systemic change management in current hospitals in the selected European countries that may jeopardize the implementation and sustainability of such changes. Increasing digitization and new technological trends are certainly driving these changes and may pose a threat of destabilizing and compromising healthcare if they are carried out without a complex (and competent) approach. We can point at the WHO guideline [6] presenting recommendations based on a critical evaluation of the evidence on emerging digital health interventions that are contributing to health system improvements, including an assessment of benefits, harms, acceptability, feasibility, resource use, and equity considerations, which links closely to Labrique AB [15]. Digital health should be an integral part of health priorities and for this purpose the Global Strategy on Digital Health 2020–2025 was initiated [14].

### Setting the Scene of Change Management Analysis in Healthcare

There are many definitions of change management with different structures, levels of detail, and support tools. Across publications, we often find Kotter’s 8-step model, Lewin´s 3-step model, or the Soft System Methodology technique, but there are also the Adkar model developed by Prosci Research, or the McKinsey 7S framework.

Although Kotter´s method is primarily known in the world of business, its core concepts can be applied also to the healthcare area. Small et al. [16] state that this approach is suitable for healthcare organizations especially because it offers a solid structure and is easily understandable by employees. Incorporating employee feedback and suggestions into the final concept of change implementation is a great benefit of this approach. Kotter [17] states that the method also allows for an early identification of potential errors, badly designed processes, and subjects not ready for the change. To see how widespread this approach is, one can just look at the high number of case studies focusing on the healthcare sector. Maclean and Vannet [18] applied Kotter’s model to introduce new diagnostic techniques based on imaging technologies and concluded that the approach is a suitable tool for increasing healthcare standards. Dolansky et al. [19] used Kotter´s model in the context of increasing treatment effectiveness in patients after heart failure. Ellsbury et al. [20] successfully applied the approach to increase outcomes for preterm newborns. One of the key activities in healthcare is the communication among various actors during provision of healthcare services. Several authors including Baloh et al. [21] or Small et al. [16] have focused their research in this direction (e.g., efficient information sharing between nurses during shift handover).

Lewin’s model is based on three steps of the change process: unfreezing, changing, and refreezing. The key idea behind Lewin’s change management model [22] is that any change for the better is not sustainable in the long term and returns to the previous state, unless the goal is clearly defined including the sustainability and consolidation of the new state. Lewin’s model may also include field theory, group dynamics and action research as applied by a number of researchers including Šuc et al. [23], who described changes in patient cost allocation in the IT system. Abd El-Shafy et al. [24] applied this approach to organizational changes, particularly in the implementation of a traumatology program. Shatpattananunt et al. [25] used Lewin’s principle to implement EBM procedures. Tetef [26] analyzed the pitfalls of new technology deployment in asthma treatment (bronchial thermoplasty). These authors concluded that the success of a change requires a clearly defined strategy, interprofessional collaboration among employees, open communication, mutual support between departments, information from facilities having previous experience with the method, and creation of educational materials.

Another frequently used method is the Soft System Methodology (SSM). SSM has been supported by the Royal Academy of Engineering, the King’s Fund and, finally, the NHS [27]. SSM rather aims at building understanding of the issue through stakeholders, but it does not represent a structured problem deconstruction [28], as there is a difference between the problematic situation and the problem [29]. According to this method, a holistic approach to the system is more efficient than splitting the system into its constituent parts, which might lead to an omission of important interactions and/or relationships between parts of the system [30]. This concept is also supported by one of Schneider’s four ideas [31] explaining why some changes succeed while other fail, namely that interventions focused on the entire system have a greater potential to succeed. The idea of a “hard” system refers to an approach that does not take into account different world views, something firmly embedded in social interactions. From the “soft” system perspective, the world constantly evolves and changes through behaviors of people [32]. “Soft” systems are characterized by complex stochastic behaviours with unknown probabilities. Such behaviors tend to be indeterminate and unpredictable [33]. This method was applied by Newell et al. [28] to implement changes in the emergency department processes when an asthmatic patient arrives, and by Mukotekwa and Carson [34] and Emes et al. [35] to improve the patient release process.

The above methods are compared in Table 1. The table indicates that Kotter´s and Lewin´s methods share a number of parameters. The SSM method shows more differences, which is mainly due to its systematic view at problem situation solution, as well as by its cyclic nature.

There are significant differences in the CHM level and the systematic approach to CHM in healthcare across countries. Requirements on the management of healthcare facilities to cope with changes are constantly increasing, especially in these turbulent times.

Available publications show that the changes occurring in the healthcare sector have shown various characteristics, but the overall trend is towards digital innovations. The process of digital transformations must be under control, well managed, and follow a clear vision. Innovative digital products come in rather short development cycles, creating a fast-changing market. New innovations require a reappraisal of internal working processes currently in place in the particular organization. Hence, it is important not only to flexibly respond to new trends, but also to create conditions for their implementation through change management [36]. The question is whether healthcare systems can be nimble enough to deal with the onset of the many digital innovations (changes). It is important to keep in mind that management skills are necessary on several levels in order to successfully accept such innovations [37]. Brice and Almond [38] studied these management skills and concluded that the design of innovations must be adapted to complex human behaviors. The difficulties with innovation acceptance was also described by Shaw and Chisholm [39]. Similarly, the OECD has called for a gradual approach to the implementation of new digital innovations [40].

## 2. Materials and Methods

This paper poses seven research questions (Table 2), using the following methods to find the answers. The answers are further presented as “findings” under the same number as the relevant question.

### 2.1. Questionnaire Survey

**Figure 1:**
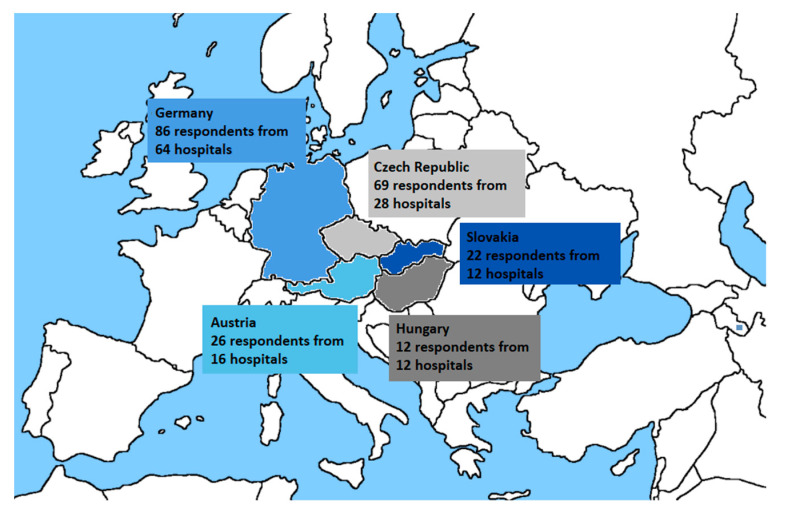
Regions analyzed: numbers of hospitals and respondents participating in the research.

When creating the questionnaire on the use of CHM in Central European countries, the first step was an identification of typical key factors that occur during change implementation in hospitals. The questionnaire structure (see Supplementary Material Table S1) was developed in collaboration with an expert from the General University Hospital in Prague, partly utilizing the assumptions of the checklists available from Prosci Research, a company providing long-term counselling in the area of change implementation [41]. Subsequently, the questionnaire was translated into two languages (English and German) and distributed in Germany, Austria, the Czech Republic, Slovakia, and Hungary (see Figure 1) using the Survio application.

In step 2, the findings of the questionnaire served as the basis for the definition of the area that the systemic research was focused on (change implementation in the context of digital innovations).

Respondents could fill out the questionnaire between 1 January 2021, and 28 February 2021. The addressed hospitals were selected based on the number of beds (addressing those with more than 500 beds). Members of hospital management such as directors, deputy directors and quality managers were approached by e-mail. Table 3 gives details on the hospitals that participated in the study.

Result processing and visualization were conducted using basic statistical methods and standard statistical tests (above all Pearson’s χ^2^ homogeneity test) in MS Excel 2013.

Based on the survey results, the most important area of CHM highlighted by the questionnaire respondents was selected for a systematic literature review: CHM in the area of digital innovations and introduction of IT procedures into hospitals. The systematic review was conducted in accordance with the PRISMA statement [46]. Table 2 lists the basic research questions addressed in this research. Questions 1–6 are related to the questionnaire survey. Question 7 is answered by the systematic review.

### 2.2. Systematic Review

A literature search focused on digital innovations and the introduction of IT procedures into hospitals was conducted on the Web of Science, PubMed, and Scopus databases in line with the PRISMA statement [46]. The keywords were as follows: “trend”, “digital”, “evolution”, “hospital”, “healthcare”, “transformation”, and “innovation”. These keywords were used to create search terms. Searches for the relevant literature were conducted from January to March 2021. The basic search criteria are shown in Table 4, and Table 5 lists the inclusion and exclusion criteria used in the search for relevant studies.

## 3. Results

### 3.1. Questionnaire

Out of 368 hospitals that met the criterion of 500+ beds in the selected countries, 132 returned filled-out questionnaires. The research team contacted several persons responsible for hospital management in each hospital; a total of 215 respondents filled out the three-part questionnaire, as detailed in Table 6. Slovakia and the Czech Republic had the highest return rate, while the Czech Republic and Germany generated the highest absolute number of responses. The smallest number of responses came from Hungary.

#### 3.1.1. Finding 1 (Research Question No. 1)

More than a half of all respondents (58%) stated that they use change management methods, while the rest do not employ CHM (31.5% stated an explicit NO, 10.5% did not give an answer). There were no differences in answers across the countries (tested by Pearson´s χ^2^ test with 4 degrees of freedom: χ^2^ = 1.603; *p* = 0.808). Those who gave a negative answer, i.e., those who do not apply CHM principles, stated personal obstacles (26%) and impracticality (14%) as the main reasons.

#### 3.1.2. Finding 2 (Research Question No. 2)

**Figure 2:**
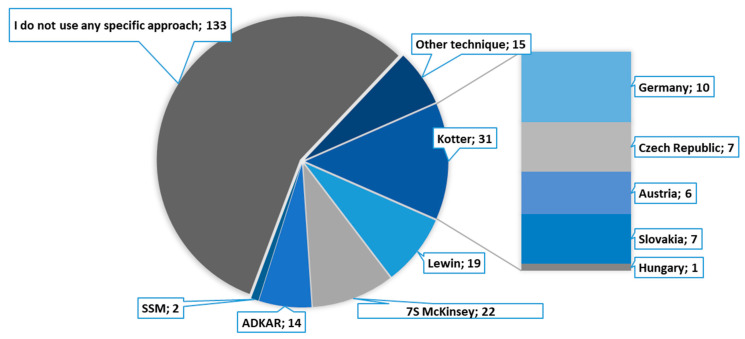
Use of particular CHM approaches in hospital management (pie chart), use of Kotter´s method by country (bar specification on the right-hand side).

A closer inquiry into which specific CHM approaches are used by the respondents showed that the best-known theoretical procedures are not used very frequently. In other words, top managers seem to realise the importance of a systemic approach to change management, however, their specific steps and measures in structuring changes are based largely on subjective considerations. This conclusion is also supported by the finding (Figure 2) that more than a half of respondents (62%) stated that they were not aware of an application of any particular CHM technique. More than a third of respondents indicated that they were aware of particular CHM approaches and used them.

According to the results, the most commonly used CHM method is Kotter´s 8-step model, but its specific use varies widely across countries. See Figure 2 for a detailed analysis of this area.

#### 3.1.3. Finding 3 (Research Question No. 3)

In terms of who is responsible for the implementation of changes in healthcare facilities, the answers from different countries varied greatly and are shown in Table 7. The term *whole team* is based on the terminology used by Kotter; it is possible that the respondents did not differentiate between *top management* and *whole team* (the questionnaire did not provide explanation on this level of detail). The respondents stated that the highest authority for change management is a member of the top management in most cases.

#### 3.1.4. Finding 4 (Research Question No. 4)

**Figure 3:**
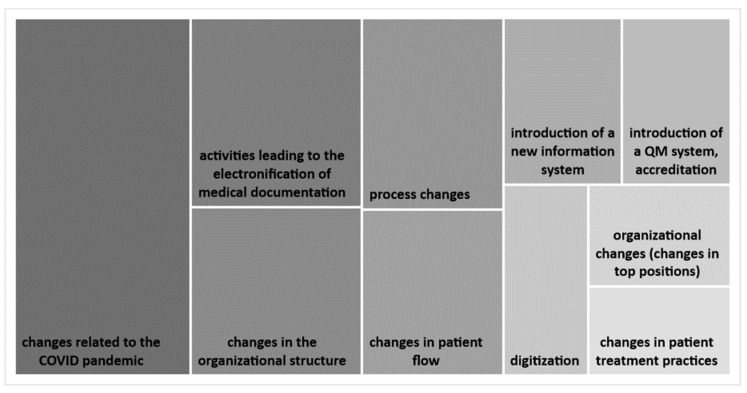
The Top 10 most common changes taking place in hospitals.

As for change typology, most respondents (81%) stated that the most common kinds of changes involve processes followed by systemic changes (12%). In a detailed open-ended question, the respondents defined a total of 57 change types. Figure 3 gives an overview of the most frequently mentioned change types. The majority of respondents (88%) indicated that the most current and novel changes involve IT and digital innovations.

#### 3.1.5. Finding 5 (Research Question No. 5)

**Figure 4:**
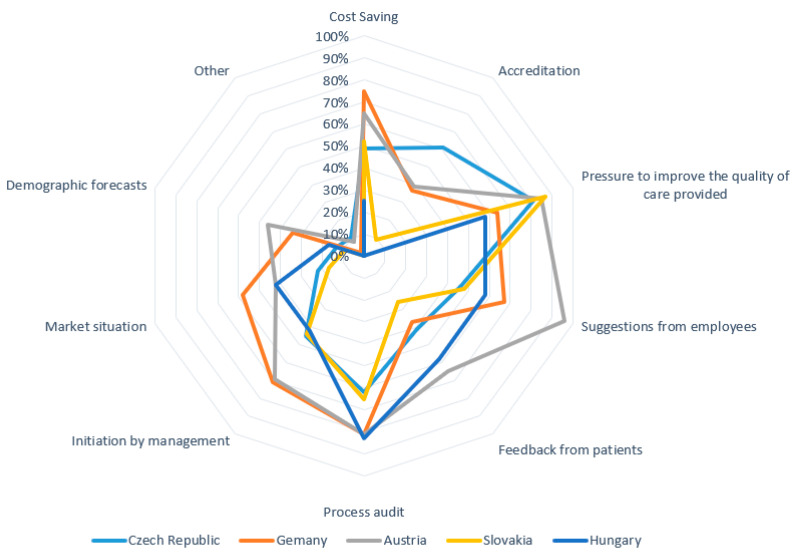
Factors leading to change implementation.

In a multiple-choice question *“what factors lead you to decide that a change is needed”*, 215 respondents gave a total of 1026 answers. Figure 4 shows a detailed distribution of the answers by country.

**Figure 5:**
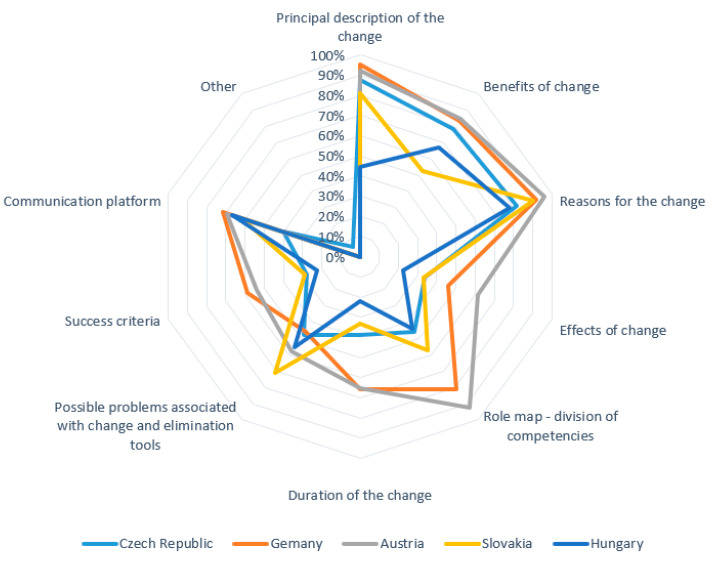
Information provided about the change to the involved persons.

Further questioning focused on whether managers have a standard strategy to manage resistance to planned changes. The results indicate that most respondents (59%) use a standard strategic approach to resistance management, but nearly a third (27%) of them do not have such a strategy. The remaining respondents (15%) chose a neutral answer. Figure 4 indicates that the Quadruple Aim is not hit in terms of priority and practice for many of the respondents or the sample as a whole. The patients’ voices are clearly not strong, and compliance and cost issues, although indicated here, do not show any clear association with the other areas of the Quadruple Aim. Managing resistance among stakeholders is the key step in the change process. The questionnaire also asked about the information that managers provide to persons involved in the change. The multiple-choice question “*what information about the change do you provide*” yielded 1181 responses (see Figure 5).

#### 3.1.6. Finding 6 (Research Question No. 6)

**Figure 6:**
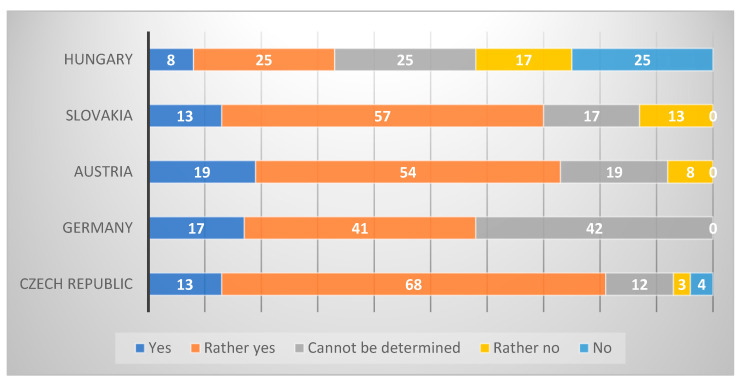
Success of change implementation by country (subjective assessment).

The questionnaire was concluded by the managers’ subjective assessment of their success in change implementations. The answers were evaluated by a Likert scale. The results (see Figure 6) indicated that most managers considered their change implementation to be successful, with only 7% conceding a failure (score 4 or 5).

**Figure 7:**
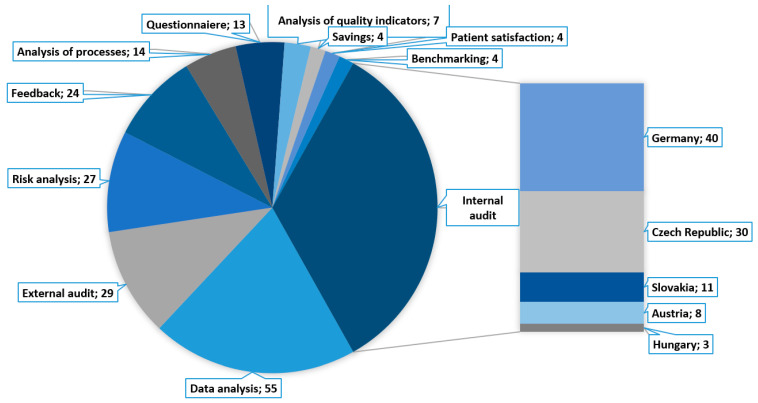
Most common approaches to change success assessment.

Finally, the questionnaire focused on what information or sources are used by managers to assess the relative success or failure of the implemented changes. This open-ended question generated 288 answers. The most frequently repeated answers are shown in Figure 7. The success of a change is most usually judged based on an internal audit (92 responses) when 40 of 86 German managers gave this answer, as well as 30 of 69 Czech managers, 11 of 22 Slovak managers, 8 of 26 Austrian managers, and 3 of 12 Hungarian managers.

### 3.2. Systematic Review

**Figure 8:**
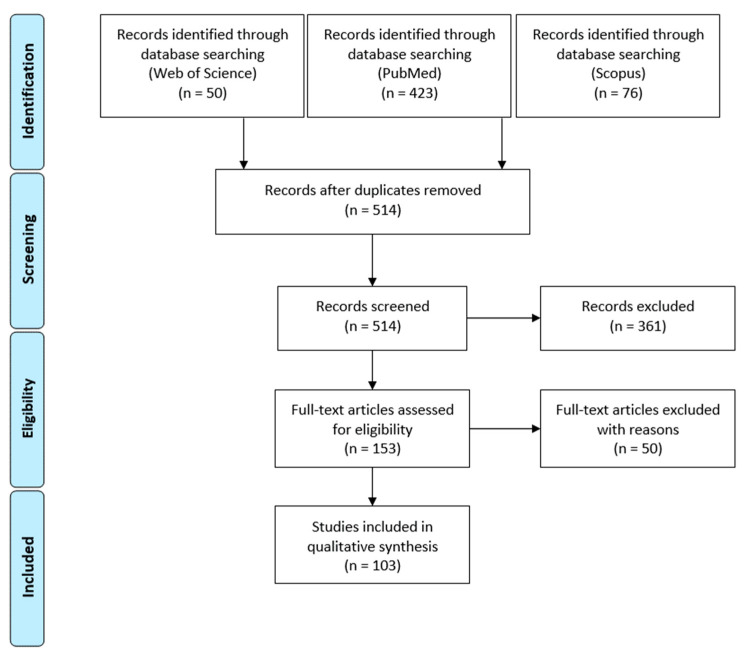
PRISMA scheme of publication selection.

The PRISMA statement scheme in Figure 8 shows that 103 research publications were selected and included in the final step of the systematic review analysis (for a detailed list of the selected publications see Supplementary Material Table S2).

**Figure 9:**
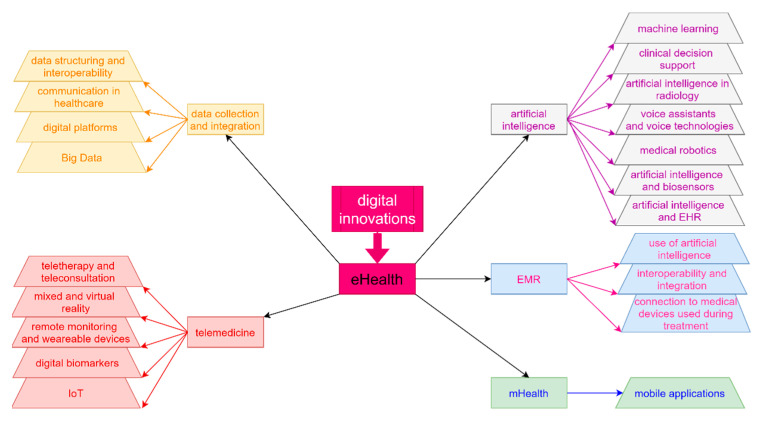
Research directions in publications on digital innovations (IoT (internet of things), AI (artificial intelligence)).

Figure 9 offers a graphic representation of the research directions found in publications in the area of digital innovations. It should be noted that the studies sometimes use inconsistent terminology in this area [47,48].

#### Finding 7 (Research Question No. 7)

Table 8 gives a summary of opportunities and threats in digital innovations in healthcare identified in our literature review.

## 4. Discussion

This study consists of two parts. In the first part, an extensive survey of the state of CHM and opinions on CHM was conducted among managers of large hospitals in five Central European countries (Czech Republic, Slovakia, Germany, Austria, and Hungary). The results show that all these countries (regardless of their historical development) deal with similar CHM challenges with a similar degree of success. The questionnaire clearly showed that there is still considerable room for improvement in terms of the use of specific CHM tools in the hospital setting. Managers understand that changes must be managed in a systematic way; however, they often rely on intuitive actions. The need for change arises from external (technological progress) but often also internal (efforts to cut costs or increase care effectiveness) causes. Managers work hard to provide information to all actors involved in the change on a regular basis, but they sometimes neglect such fundamental things as success criteria, potential problems they might encounter during change implementations, and/or how to deal with these problems.

As many as 86% of respondents indicated that the most contentious area of changes is the implementation of changes in IT and digital innovations. Based on this information, a systematic review was conducted in the second part of the study in order to identify literature sources that address CHM use in healthcare digitization and electronization. The literature search yielded 106 research papers that offer a comprehensive overview of application of CHM methods in healthcare, the relative success of their implementation, and problems encountered in CHM (for the full list of selected papers see Supplementary Material Table S2).

Change Management is extremely topical at present in COVID-19 times, when a lot of temporary or permanent changes have had to be implemented in hospitals (and healthcare systems generally) all over the world. However, we did not concentrate on any particular time-specific changes or episodes. The paper aims to find general rules. Of course, they are beneficial for COVID-19-induced changes as well.

Apart from showing regional specificities, the findings of our study support the results published in plentiful literary sources. We can see that efforts to enforce changes in a top-down manner are rarely met with enthusiasm from front-line healthcare professionals. Healthcare systems are too complex for such an approach, and front-line workers are not ready to linearly respond to changes initiated purely from the top [114,115]. A bottom-up approach has been supported, for example, by Kraus et al. [116]. Continuous improvements of a healthcare system require real-time feedback [114], opportunities for short-term victories [116], and, at the same time, training for healthcare staff in systemic thinking, knowledge, and the skills necessary for the implementation of changes [114,117]. The need for the involvement of employees is also highlighted by the Scandinavian model presented by Nielsen et al. [118], which requires cooperation between employer and employees. Nielsen and his colleagues believe that the importance of the employees’ role in innovation development and implementation is generally overlooked in the entire eHealth area [119]. Guy et al. [81] offers a case study of efficient employee engagement in a change process where an augmented reality machine was introduced to a workplace. However, a problem may arise if the actors do not share the same goals or expectations concerning the innovation process [106,120]. This may result in a confrontation between management’s vision and the experience of first-line workers who directly use the innovative technology in their work, i.e., a “clash of dreams and reality” [106,121].

No one should expect that an innovation will be easily accepted and deliver the expected benefits simply because it has been developed [106,122]. Organizational preparedness is equally important, including, among other things, motivation (need for change), resources (infrastructure), employee attitudes (adaptability), and organizational climate (goal clarity) [37,106]. The perception of a need for change may also be affected by whether the innovation is seen as an opportunity or as a threat [106,123]. Employee adaptability also depends on how the innovation will change employee roles and work procedures. Rigorous management is especially indispensable in situations when a change has a major impact and presents a sensitive issue [106]. Setting national laws to enforce mandatory use of an IT system has a substantial effect on the adoption of such a system, as has been proven in the case of outpatient electronic prescription [124].

As our systematic review shows, while research in AI has been meeting with ever growing interest, the practical impact of the technology applications in real-life settings has received much less attention. Unless AI is introduced gradually, long-term problems with and/or for employees may follow [104]. The innovation potential of AI may lead to substantial changes in organizational procedures, and the management must consider the impact on all stakeholders and align with their priorities [104]. Examples of potential obstacles include lack of engagement among patients/users, resistance among healthcare workers, insufficient networks and processes, economic and legal factors, lack of political support, lack of knowledge, and safety and data protection [94,125].

Research by Muffly et al. [126] found that more than 70% of physicians state that they have experienced some level of stress in relation to healthcare information technologies [62]. According to Lo et al. [62], assessment of the success in an implementation should take into account the impact on the mental health of employees. Management will have to deal with challenges in driving change acceptance, including problems related to infrastructure, data integration, interoperability, and safety [127]. The circumstances under which eHealth elements are introduced, therefore, require a holistic approach. In other words, organizational processes and structures, human resources, education, legislative requirements, and other factors must be adapted for these purposes [52]. It is important to arrive at a shared point of view and align the needs of all stakeholders [102,128]. It is equally important to anticipate and adequately manage certain resistance from the part of employees [102,129]. This resistance is a natural phenomenon that also arises in other industries; people have a natural aversion and resistance to change [52].

Hermes et al. [67] identified several reasons why the healthcare sector is slower in adopting new technologies. First, healthcare is a complex sector with many interconnected stakeholders that is strictly regulated by governments. This leads to a lack of interoperability among the stakeholders [67,130]. Other factors include healthcare workers’ unwillingness to learn new things, an unfavorable environment where medical authorities oppose innovation and exert influence over other physicians, fear of losing physicians’ autonomy, fears linked to safety and privacy, initial and ongoing costs, technical issues, loss of productivity during the change implementation, and concerns of future obsolescence [67]. Innovation acceptance may also be affected by patients themselves who increasingly care for the confidentiality of their personal data and are less and less willing to share their data [67,131]. Patient concerns have been confirmed by a systematic review by Kraus et al. [116] who also state that the process of change acceptance is further slowed down by professionals’ reluctance to give more power to patients, especially due to data reliability concerns. Naidoo [132] emphasizes that the biggest obstacles will not be of a technical nature but rather a social one. At times the obstacles also come directly from the supplier of the innovation who fails to create a user-friendly or reliable product [67].

McKinsey, in collaboration with the World Economic Forum, concluded that the biggest recent challenge has not been innovation development, but innovation implementation [102,133]. At the same time, as customers, in this case patients, acquire a deeper understanding of the concept of “correct” patient-centered care, they will be a powerful factor in driving changes [102,134]. Changes often seem to appear out of the blue: although stakeholders are distracted daily by efforts to make the system more and more suitable for their needs, they may be surprised by a new challenge, as it may come from an unexpected source and bring about hitherto unimagined ways of doing things. Our understanding is limited by concepts that we can put into words [100].

There are some factors that can be controlled and might have a positive impact on the result of change implementation: above all, leading and managing with a clear vision, engaging all stakeholders early and with clear rules, setting up communication channels and strategies, adapting to the local context, providing a technical base, proceeding step by step, and regularly seeking feedback including monitoring [102,129,135,136].

Proving the benefits of digital innovation is usually much simpler than implementing such an innovation. For example, the benefits of Anesthesia Information Systems (AIMS) were documented twenty years ago [137], but only 75% of academic hospitals in the USA had AIMS in 2014 [138]. Despite possible cost savings, initial lack of funds for IT is often seen as the most substantial obstacle [139]. Moreover, particular projects are only singularly followed up in the literature. Hence, for a foreign author (as a person without local knowledge), it is usually not feasible to properly assess the genuine outcome of an intended innovation.

Our research has several limitations. The questionnaire targeted hospitals in five Central European countries, i.e., findings and conclusions may not be easily generalized for the entire European area. The rate of return of questionnaires was rather limited, especially in some of the involved countries (despite a three-round system). On the other hand, the results of this study offer a realistic picture of the current CHM situation in the region and provide a valuable basis for potential further work in this area. Unfortunately, Poland could not be included, as communication with Polish authorities and a search in relevant sources revealed that Poland does not have a central system registering Polish hospitals that includes data on the number of beds, the key criterion for a hospital’s inclusion in the questionnaire study.

We assume that more qualitative research needs to be done in this area focusing on the motivation and attitudes of hospital management to a fast-changing environment that includes changes arising due to an enormous growth of digital innovations. Another key question with considerable research potential is the issue of the sustainability of changes in the current healthcare environment.

## 5. Conclusions

The healthcare sector is a complex environment, so introducing technological and digital innovations requires careful and well-designed change management. As healthcare (similar to other sectors) is becoming increasingly dependent on digital innovations, be it in terms of treatment efficiency, or communication and diagnostic optimization, change management is gaining importance in this area [97]. Change planning, evaluation, and management is, therefore, equally as important as the innovations themselves, since these processes help secure the desired results and benefits [65].

In light of the changes that will be brought about by Industry 4.0 and digital healthcare innovations, many opportunities as well as threats present themselves loudly. The CHM literacy needs to be strengthened, and experience and best practices need to be shared across hospitals and regions.

The main factors to be monitored for a successful and sustainable CHM include a clearly defined and widely communicated vision, early engagement of all stakeholders, clearly set rules, adaptation to the local context and culture, provision of a technical base, and a step-by-step implementation with strong feedback.

This paper may contribute to planning and implementing future changes by a detailed description of the current situations, problems, and benefits in Central European countries (with different backgrounds). The review provides a synthesis of experience in changes concerning the introduction of digital innovations in healthcare.

## Figures and Tables

**Table 1:** Comparing basic characteristics of selected CHM methods.

Field	Lewin	Kotter	SSM
External expert	maybe	maybe	no
Pre-defined problem	yes	yes	no
Feedback	yes	yes	yes
Sociological approach	positivism	combination	interpretivism
Support tools	field theory, action research, group dynamics	“see-feel-change”	CATWOE, PQR, “root definitions”
Dismantling existing situation	in step 1	in step 1	during the process
Cyclical process (constant problem solving)	yes	yes	yes
Management/leading coalition	yes	yes	no
Recommended step sequence	yes	yes	steps overlap in reality
Building awareness why the change is necessary	yes	yes	not necessary, awareness arises spontaneously
Adaptability to changing environment	no	partly	yes
Suitable application	smaller changes	bigger changes	complex problems
CHM approach	top-down	top-down, partly bottom-up	combination of bottom-up and top-down

**Table 2:** Key research questions.

Question No.	Key Research Question
1.	Do the majority of healthcare managers use CHM tools?
2.	What specific CHM method do healthcare managers use the most?
3.	What hospital actors are most involved in change implementation and management?
4.	What changes (of what type) are currently most frequently being implemented in hospitals?
5.	What is the target of the change implementation and what information do mangers provide to those involved in the change?
6.	What do managers think about the implemented change? How do they judge the success/failure of a change?
7.	What opportunities and threats can be identified in the context of digital innovations?

**Table 3:** Specification of the hospital sample [42,43,44,45].

Country	Population ^1^	Beds Per 100,000 Inhabitants (Rounded off) ^2^	Number of Hospitals with 500+ Beds ^3^	Number of Participating Hospitals
Czech Republic	10,693,861	662	36	28
Germany	83,135,181	800	244	64
Austria	8,904,262	727	26	16
Poland ^3^	37,941,122	654	n/a	n/a
Hungary	9,771,975	701	44	12
Slovakia	5,457,679	570	18	12
In total			368	132

^1^ Eurostat data as of 2020. ^2^ OECD data as of 2019. ^3^ These data were collected from sources [42,43,44,45] and clarified through personal correspondence.

**Table 4:** The strategy of database searches.

	Web of Science	PubMed	Scopus
Search terms	(((“trend”[Title] OR “evolution”[Title] OR “digital”)[Title]) AND ((“hospital”[Title] OR “healthcare”)[Title])) AND ((“transformation”[Topic] OR “innovation”)[Topic])	(((“trend”[Title] OR “evolution”[Title] OR “digital”)[Title]) AND ((“hospital”[Title] OR “healthcare”)[Title])) AND ((“transformation”[Title/Abstract] OR “innovation”)[Title/Abstract])	TITLE (“trend”OR “evolution” OR “digital”) AND Title (“hospital” OR “healthcare”) AND TitleABS (“transformation” OR “innovation”)
Time period	2018–2020	2018–2020 (6 December 2020)	2018–2021 (7 Mar 2021)
Languages	English, German	English, German	English, German
Document type	Papers, conferences, reviews, editorial material, early access	Papers, conferences, reviews, case studies, clinical trials, systematic literature review, randomized controlled trials	Papers, conferences, reviews

**Table 5:** Inclusion and exclusion criteria used in the systematic literature review.

Question	Inclusion Criteria	Exclusion Criteria
Does the study focus on the use of digital innovations in healthcare?	implementation or use of digital innovation as the main topic; digital innovation in any healthcare intervention with elements of e-Health, virtual reality, smart phones/portable devices, or telemedicine as part of its implementation; aimed at understanding why the innovation is being incorporated into healthcare activities	the main topic focuses on the creation of measures, checklists or other metrics that do not represent a healthcare digitisation intervention; merely a technical description of an innovation; studies focusing on safety, education, or ethical issues related to digital innovations
Does the study deal with the framework for a digital innovation implementation?		studies focusing on theoretical mathematical models or statistical models and simulations of
Is the study rooted in the hospital environment?	hospital or clinic environment	other environments (e.g., pharmaceutical companies, medical device manufacturers)

**Table 6:** Questionnaire return rate in participating EU countries.

Country	Number of Potential Respondents Contacted	Number of Answers	Return Rate
Czech Republic	279	69	26%
Germany	1389	86	7%
Austria	293	26	10%
Slovakia	78	22	30%
Hungary	298	12	4%
Total	2337	215	11%

**Table 7:** Who is responsible for change implementation and management in the hospital.

	Top Management	Whole Team (Leadership Coalition)	Middle Management	Quality Managers	Project Managers	HR Dept.	Outsourcing	Other
Czech Republic	70%	32%	19%	33%	9%	6%	1%	6%
Germany	46%	71%	40%	27%	31%	6%	2%	1%
Austria	35%	77%	31%	15%	12%	4%	4%	4%
Slovakia	65%	17%	0%	26%	4%	0%	0%	9%
Hungary	67%	33%	17%	8%	8%	0%	0%	0%
% (average)	48%	51%	27%	26%	17%	5%	2%	4%
Absolute number	104	110	57	56	37	10	4	8

**Table 8:** Opportunities and threats for digital innovations.

Technology	Opportunity	Threat
mHealth	Wide user basis of mobile phone users [49,50] Rapid growth in the number of applications supporting self-management [51,52,53] Applicable to a wide scope of diagnoses [47,53] Increased patient engagement during treatment [47,52,53,54,55]	Ethical and legal aspects [53,56,57,58] Limited evidence of outcomes and benefits (insufficient randomised controlled trials) [47,52,56,59,60] Low interoperability and integration with existing work procedures [56] Uncertainty concerning data reliability [47,56] Declining patient self-discipline over time [52] Absence of personal contact with physician [55] Non-certified applications, large number of applications [61,62] Level of physician acceptance of mobile health applications [62]
Electronic Health Record (EHR), Electronic Medical Record (EMR), Personal Health Record (PHR)	Access to information for all stakeholders [63,64,65,66,67] Benefits if combined with AI [58,65,68] Higher accuracy, legibility, reliability, and better information search functions [64,65,69,70] Risk management—reminders, warnings (allergies, patient history) [64,67,70] Less burden on treating medical staff [36,64] Reduction of cost related to poor documentation [64,65,69]	Violation of the interoperability condition [53,63,70,71] Problem with aligning operating standards with the current information exchange protocols for Big Data [72] Regulatory restraints [72,73,74] The risk of possible re-identification [74] Financial sustainability [75]
Digital biomarkers	Wide user base [76] Wide range of information [76] Better diagnostic and decision-making on interventions thanks to continual data collection [58,59] Developing flexible electronic materials for integrating chip technology [77,78]	Bad choice of monitored attributes [59] Problems with technology validation [59]
Telemedicine	Lower risk of disease transmission [79,80,81] Suitable for “social distancing” [82] Reduction in hospitalization cost [83,84] Comparable or better care than that of in-person consultations [79,83,85] Elimination of the feeling of isolation during hospitalization [79] Alleviation of resource scarcity (staff, geographical location) [84,86,87,88] Shorter waiting times [60,86] Applicable to numerous diagnoses (e.g., in psychiatry, dermatology, etc.) [60,89,90,91,92]	Limited applicability based on diagnosis [79,85] Unreliable Internet connection [79,85] Lack of training in the use of digital devices [60,79,93] Violation of interoperability between healthcare providers and healthcare systems [94] Discrimination of certain patient groups (e.g., people with particular handicaps) [80] Limited evidence of outcomes and benefits (insufficient randomised controlled trials) [60,80]
Artificial intelligence (AI)	Prediction of illness development [94,95,96,97,98] Improvements in treatment optimization and effectiveness [94,97,99,100] Evidence-based recommendations [60,98,101] Delegation of simple and repeating tasks to AI [96] Lower number of hospitalizations [95] Cost cutting [77,95,97] Less pressure on scarce HR in healthcare [102,103] Automatic recall and rescheduling of patients [98] Bigger potential of other digital innovations [68,104] Ability to process huge amounts of data [101] AI-biosensors (miniaturization, scalability, low power consumption, high sensitivity, multifunction, safety, non-toxicity, and degradation) [77]	Incompatible with older infrastructure [105] Lack of understanding of AI functionality [68,106] Inefficient use of AI in day-to-day workflows [107,108] Potential conflict between human ability to act autonomously and the complicated, allegedly infallible machine logic (known as automation bias [69,100] Legal and ethical issues [68,95,100,101,104] Physicians’ concern about AI (security, privacy, and confidentiality) [68,101] Missing multidisciplinary AI teams [98]
Wearable technologies	Wide user base [76,77,93] Better diagnostics and decision-making about interventions thanks to continual data collection [76,77,91,106,109] Source of objective data (measured in real-life conditions) [76,91,110] Reduction of “unnecessary” out-patient visits [94] 4P medicine (predictive, precise, preventative and personalized) [76,77,111]	Data smog [76,91] Standardization and validation issues with sensor placement [91] Energy consumption (limited battery capacity) [49,84] Different levels of digital literacy and/or aproach to technologies among patients [47,91] Declining patient self-discipline over time [91] Limited availability due to high production costs of some technologies [77]
Internet of Things (IoT)	Higher operational efficiency [49,112] Integration of data from various sources [49,112] Disease prevention and monitoring [49,113] Use of AI in analyses [49,65,113]	Loss of safe and stable communication with devices [84] Higher demands on network infrastructure [49] Unauthorised manipulation [49,112] There are currently no clear instructions for healthcare staff how to use IoT (e.g., in recommendations to patients concerning their use) [49]

## Data Availability

The data presented in this study are available in the Supplementary File.

## Supplementary Materials

The following are available online at https://www.mdpi.com/article/10.3390/healthcare9111508/s1, Table S1: The questionnaire structure; Table S2: Detailed description of articles from the PRISMA guidelines.
