# Supplementary material for: Change Management and Digital Innovations in Hospitals of Five European Countries
Source: Healthcare (Basel). 2021 Nov 5;9(11):1508. doi: 10.3390/healthcare9111508 (PMC8625074; doi:10.3390/healthcare9111508)
Supplement: Supplementary file 1 [file healthcare-09-01508-s001.zip › Table S1.pdf]

healthcare

MDPI

Table S1. The questionnaire structure.

Questions in English	Answers in English
What is Your job title in the hospital ?	Director<br>Deputy for Medical Care<br>Deputy for Nursing Care<br>Technical and operational deputy<br>Quality manager<br>Project manager<br>Crisis manager<br>Personnel department<br>IT employee<br>Matron<br>Superintendent<br>Other
Do you use structured change management in your hospital?	Yes, no, I don't know
What is the reason for not implementing change management?	We don't consider it necessary, personnel reasons, financial reasons, I don't know, other
Who is in charge of implementing the changes in your hospital?	Top management<br>The whole team (so-called "leading coalition")<br>Middle management<br>Quality managers<br>Project managers<br>Personnel department<br>External company (outsourcing)<br>Other
Is the highest authority always someone from the top management?	Yes, no
What typology of changes is the most common in your hospital?	System changes, process changes, I don't know
Please briefly specify some concrete changes that have taken place in your hospital.	*Write in your answer please*
Do you consider the changes in IT and digital innovation up to date?	Yes, no
Do you adjust the approach (and therefore the methods) to the changes of the organizational culture and nature of the change?	Yes, no
Which specific method of change management do you incline to while implementing changes?	I am not aware of using any of the methods<br>Lewin's method<br>Kotter's method<br>ADKAR<br>7S McKinsey<br>Soft System Methodology<br>other

What is the need for change based on in your opinion?	Cost savings<br>Accreditation<br>Improving the quality of care provided<br>Propositions from employees<br>Propositions from patients<br>Analyzing processes<br>Management's initiative<br>Market situation<br>Demographic prognosis
Do you discuss the change with individuals who are going through it?	Yes, no
What information do you provide about the change?	What will the change be based on<br>What will we gain from the change<br>The reasons why the change is needed<br>What will not change<br>Role map - who would do what<br>How long will it take<br>Problems - what difficulties will we face, how to deal with them<br>Criteria of success - how and on what basis we recognize success<br>Communication channels - how will the people be informed
Do you create space for interested parties' feedback?	Yes, no
Do you have any established strategies to manage the interested parties' opposition to your change?	Yes, no
Are you successful in implementing changes? Use the Likert scale to demonstrate the successful rate of implementing changes.	Yes, rather yes, cannot be determined, rather no, no
On what basis do you determine the success of the changes? (e.g. internal audits, data analysis, risk analysis, etc ...)	*Write in your answer please*

Request for filling in the questionnaire "Change management in hospitals"

Dear participants,

Thank you for your willingness to spend time with the questionnaire regarding change management in hospitals. Answering the questionnaire should not take more than 5-8 minutes. Most of the options are in the form of checkboxes.

Please, be informed the process of data collecting is completely anonymous.

Your inputs will help me with elaborating the thesis.

Should you have any questions, do not hesitate to contact me.

P.S.: In case you know about more competent person in your organisation, I kindly ask you to forward it. Thank you.

Reminder: Invitation for a short questionnaire "Change management in hospitals"

Dear participants,

May I kindly remind you to complete the questionnaire aimed for my thesis data collecting.

Your participation will help me with gathering valuable data.

Thank you for your time and provided answers.

Should you have any questions, do not hesitate to contact me.

P.S.: In case you know about more competent person in your organisation, I kindly ask you to forward it. Thank you.
