# Supplementary material for: Change Management and Digital Innovations in Hospitals of Five European Countries
Source: Healthcare (Basel). 2021 Nov 5;9(11):1508. doi: 10.3390/healthcare9111508 (PMC8625074; doi:10.3390/healthcare9111508)
Supplement: Supplementary file 1 [file healthcare-09-01508-s001.zip › Table S2.pdf]

healthcare

MDPI

Table S2. Detailed description of articles from the PRISMA guidelines.

Nr	Title	Author	Year	Article type	Destination	Methods	Goals	Findings/Summary
1	Technologies Trend towards 5G Network for Smart Health-Care Using IoT: A Review	AHAD Abdul et col.	2020	review	n/a	Literature review	to deliver a review of 5G smart health-care with a different perspective (A taxonomy for smart health-care, covering communications technologies, network types, services, application, requirements, and characteristics. Different scenarios for 5G smart health-care and its requirements. Key enabling technologies to achieve the requirements of 5G smart health-care and open issues and challenges)	A comprehensive review of 5G assisted smart health-care solutions in IoT was presented. A structure for smart health-care in 5G by categorizing and classifying existing literature was explored. A key requirements for successful deployment of smart health-care systems for certain scenarios in 5Gwere presented.
2	Organizational readiness for artificial intelligence in health care: insights for decision-making and practice	ALAMI Hassane et col.	2020	review	n/a	Literature review	to bring forward the importance of studying organizational readiness to integrate AI into health care delivery	As an important step to ensure successful integration of AI and avoid unnecessary investments and costly failures, better consideration should be given to: (1) Needs and added-value assessment; (2) Workplace readiness: stakeholder acceptance and engagement; (3) Technology-organization alignment assessment and (4) Business plan: financing and investments. In summary, decision-makers and technology promoters should better address the complexity of AI and understand the systemic challenges raised by its implementation in healthcare organizations and systems.

3	Conceptualising Artificial Intelligence as a Digital Healthcare Innovation: An Introductory Review	ARORA Anmol et col.	2020	review	n/a	Literature review	It recognised management literature to conceptualise AI as a digital innovation, referring to established uses within healthcare.	AI has the potential to improve healthcare delivery through altering clinical practice as well as optimising workflows. As an innovation, AI exhibits three key characteristics: it is self-referential, reprogrammable and capable of pronounced generativity. AI is able to potentiate innovations which preceded it, using Internet of Things, digitisation of patient records and genetic data as data sources. There are a number of potential barriers which may delay adoption of emerging AI technologies and several risks associated with their use (It is difficult to allocate sufficient IT system investment to hospitals when there are competing demands for equipment and staff,public acceptance…)
4	Metadata Concepts for Advancing the Use of Digital Health Technologies in Clinical Research	BADAWY Reham et col.	2019	original research	USA	Metadata set design based on expert consensus (33 members)<br>- Virtual meetings<br>- proposed metadata set in the form of a multi-hierarchical tree diagram (mind map)	to propose a metadata set that reflects regulatory guidelines and that can serve as a conceptual map to (1) inform researchers on the metadata they should collect in digital health studies, aiming to increase the interpretability and exchangeability of their data, and (2) direct standard development organizations on how to extend their existing standards to incorporate digital health technologies.	The proposed metadata set is informed by existing standards pertaining to clinical trials and medical devices, in addition to existing schemas that have supported digital health technology studies. It illustrates this specifically in the context of Parkinson's disease, as a model for a wide range of other chronic conditions for which remote monitoring would be useful in both care and science.
5	An Intelligent Framework using Disruptive Technologies for COVID-19 analysis	ABDEL - BASSET Mohamed	2021	original research/Case study	Egypt	Design of framework using disruptive technologies for COVID-19 analysis<br>Case study - validation	to aid healthcare team to detect the plan of treatment remotely and safely	The disruptive technologies can replace the traditional methods in the healthcare system of diagnosis, direct communication between patient and healthcare team, overcrowding in hospitals, shortage of PPE, etc. with intelligent solutions. The use of AI, IoT, and IoMT technologies via 5G and cloud platforms promote the healthcare system to perform day-to-day routine remotely. The remote services take a large burden from healthcare teams and patients, leading to the limit of

								the spread of COVID-19. The diagnosis can be remotely detected according to data captured to take the decision to treat patients in-home quarantine, hospitalized, or identify or treat patients with typical cold or flu
6	Digital Health: Opportunities and Challenges to Develop the Next-Generation Technology-Enabled Models of Cardiovascular Care	BHAVNANI, Sanjeev P.	2020	Review	n/a	Literature review	to present the challenges in implementing digital health technology and discusses how patient-centered digital health programs are designed within real-world models of remote monitoring. It also provides a framework for developing new devices and wearables for the next generation of data-driven, technology-enabled cardiovascular care	Patient-centric co-designs is an important implementation factor for how new devices are effectively used by patients and their caregivers in home-based environments. Remote patient monitoring and the acquisition of patient-generated data represents a new approach upon which physiologic trends are measured and assessed within the continuity of care. Analytic approaches such as artificial intelligence and machine learning are under development to produce new "electronic phenotypes" of cardiovascular disease and to translate these phenotypes within an iterative process that merges digital health data and dynamic clinical decision support systems for individualized cardiovascular risk stratification.
7	Virtual Care 2.0—a Vision for the Future of Data-Driven Technology-Enabled Healthcare	BHAVNANI, Sanjeev P. a Amy M. SITAPATI	2019	Perspective, opinion, and commentary	n/a	review, synthesis	Given the multitude of such requirements, embracing new technologies as a core competency of a modern healthcare organization introduces a number of critical considerations, including the following: (1) what are the organizational factors that guide the development of new digital health clinical programs?; (2) how are new technologies and data analytics integrated to actually improve population health outcomes and the quality of care?; and (3) what type of infrastructure is required to deliver care that is virtual, timely, and effective?	the recommendations for:<br>- implementation new digital health technologies<br>- creation of dynamic clinical decision support<br>was performed
8	Treating Hematologic Malignancies	BINDER, Adam F., et al.	2020	Review	n/a	Literature review	to examine the benefits, barriers and considerations for selcted components of decentralized care telehealth visits, home	Selected tools (telehealth visits, home-based care, and remote patient monitoring) can be utilized across the spectrum of

	During a Pandemic: Utilizing Telehealth and Digital Technology to Optimize Care						based care, and remote patient monitoring) with a focus on how they care important in providing appropriate care during the pandemic and how they can become integrated into standard of care in the future	care from, prevention to palliation, to reduce the care burden as well as decrease exposure risk for patients and health care teams. Decentralization of care can provide a patient-centered approach that follows a precisionmedicine algorithmwith an extra feature: providing the right treatment, to the right patient, at the right time and in the right place.
9	Do-It-Yourself Automated Insulin Delivery: A Leading Example of the Democratization of Medicine	BURNSIDE, Mercedes, et al.	2020	Review	n/a	Literature review	to examine data sharing for algorithm development and refinement, for sharing of the open-source algorithm codes online, for peer to peer support, and sharing with medical and scientific communities	Do it yourself AID systems have no regulatory approval raising safety concerns as well as medico-legal and ethical implications for healthcare professionals. Democratization of healthcare promises better health access for all. It is recognized the limitations of DIY AID as it exists presently.
10	The Price of Artificial Intelligence	COIERA, Enrico	2019	Review	Australia	Literature review	to review the cost that may need to be paid for benefits of AI, including changes in the way healthcare is practiced, patients are engaged, medical records are created, and work is reimbursed	Whilst AI will be applied to classic pattern recognition tasks like diagnosis or treatment recommendation, it is likely to be as disruptive to clinical work as it is to care delivery. Digital scribe systems that use AI to automatically create electronic health records promise great efficiency for clinicians but may lead to potentially very different types of clinical records and workflows. In disciplines like radiology, AI is likely to see image interpretation become an automated process with diminishing human engagement. Primary care is also being disrupted by AI-enabled services that automate triage, along with services such as telemedical consultations. This altered future may necessarily see an economic change where clinicians are increasingly reimbursed for value, and AI is reimbursed at a much lower cost for volume.

11	Digitalisierungsstrategien für das Krankenhaus der Zukunft/Strategies for digitalizing the hospital of the future	DEITERS, W.; BURMANN, A.; MEISTER, S.	2018	Review	Germany	Literature review	To discuss the importance of digital innovation in healthcare	Digitalization means understanding the digital maturity level of an enterprise and the digital skills of the employees. Besides an investment in products, a successful digitalization process also necessitates consideration of the cost to release employees from their obligations in order to contribute to the process as well as for a dedicated and continuing staff training and education program. While digitalization in Germany is underdeveloped regarding both the national health infrastructure and the level of digitalization in hospitals, German clinics are recognizing more and more the importance of digitalization and are initiating digitalization strategies. In this regard, it is important to learn from the European partners, who have partially established advanced and innovative solutions.
12	Localisation, Personalisation and Delivery of Best Practice Guidelines on an Integrated Care and Cure Cloud Architecture: The C3- Cloud Approach to Managing Multimorbidity	DESPOTOU, George, et al.	2020	original research	n/a	C3-Cloud has adopted a coproduction approach to developing unified multimorbidity guidelines, by collating and reconciling best practice guidelines for each condition. Clinical and technical teams at pilot sites and the C3-Cloud consortium worked in tandem to create the specification and technical implementation	to present the co-production, and implementation approach adopted in C3-Cloud, which delivers an infrastructure able to offer multiple personalize-able best practice guidelines for multimorbidity. C3-Cloud also incorporates localization, necessary to be deployed in the heterogeneous pilot site environments.	C3-Cloud had deployed collated and reconciled guidelines on multimorbidity in three European pilot sites. Achieving this required a co-production process involving the pilot site clinical teams, the C3-Cloud clinical reference group, the pilot site technical teams, and the C3-Cloud technical team. The process resulted in over 60 clinical scenarios, 300 CDS rules and over 380 design requirements.
13	Digital health for COPD care: the current state of play	DING, Hang, et al.	2019	Review	n/a	Literature Review	To highlight the evidence base of digital health approaches for COPD care. To reviewe some typical studies and outcomes along the pathway from (I) ongoing self-management of stable COPD at home, (II) in-hospital care for acute illness and conditions under medical treatment, and	

							(III) shortterm post-discharge care programs for recovery through clinical follow-up, education and training, and (IV) hospital at home programs for moderate acute conditions and early discharge	
14	New Value for a New Movement	DOHAN, Michael S., et al.	2020	review	n/a	short review - special issue	to define movements that can be characterized by its referent theories and available technologies and their applications, as well as the issues and difficulties experienced by patients, care providers, and other key stakeholders.	3 movement were defined:<br>- telehealth and telemedicine<br>- to evaluate the ability of technologies to benefit outcomes that are critical to healthcare<br>- movement associated with the concept of digital transformation,
15	A collaborative platform for management of chronic diseases via guideline-driven individualized care plans	ERTURKMEN, Gokce B. Laleci, et al.	2019	original research			to present a method and corresponding implementation of a semi-automatic care plan management tool, integrated with clinical decision support services which can seamlessly access and assess the electronic health records (EHRs) of the patient in comparison with evidence based clinical guidelines to suggest personalized recommendations for goals and interventions to be added to the individualized care plans.	The two front-end facing components, C3DP and PEP, have benefited from feedback for improvements during the usability and application testing. Unstructured feedback, expressed through the think-aloud method by the test participants, has been particularly useful. Development teams have responded to the feedback received and incorporated them in relevant tasks, such as development, deployment and training.
16	Pursuing Sustainability for Healthcare through Digital Platforms	FAGGINI, Marisa, et al.	2019	original research	Italy	the theoretical meta-model of interaction types, which inspired the prototype digital platform dedicated to the online delivery of health-related services	Due to under-investigation of several of the sustainability-related challenges, this paper aims to better understand the system dynamics that, supported by some digital enablers (eg, digital technologies and platforms), boost the sustainability of complex service systems such as healthcare.	Thus, this study contributes to advancing the systemic approach to the sustainability of healthcare systems, underlining how digital platforms, acting as multifaceted intermediaries of interactions, can boost the exchange of resources among the different actors or the active entities that populate this service system.
17	Digital health interventions in children with asthma	FERRANTE, Giuliana, et al.	2021	review	n/a	Literature Review	to evaluate the published literature examining digital health interventions for pae-	The increased use of health digital devices will likely become a relevant aspect of a proactive asthma care model in the next

							diatric asthma and explores the most relevant issues affecting their implementation in practice and the associated evidence gaps, research limitations and future research perspectives.	few years. Extrapolated data will allow physicians to provide personalized tools, tailored solutions to improve child health, improve symptom reports and appropriate specialty referrals. Before such an approach can be widely integrated in routine clinical practice, the acceptability and feasibility should be ascertained among all patients, including those disproportionately affected by the disease. Indeed, most tools have not been designed to address barriers faced by racial/ethnic minority groups or those of low socioeconomic status and poor health literacy.
18	E-Health und die Realität–Was sehen wir heute schon in der Klinik?	GEHRING, H.; RACKEBRANDT, K.; IMHOFF, M.	2018	review	n/a	Literature review	to present the current reality in the field of digitization and e-health in healthcare with perspectives for further development from the perspective of the healthcare professional	attention is focused on clinical application and interaction between users and digital technology
19	Open Innovation in Digital Healthcare: Users' Discrimination between Certified and Non-Certified mHealth Applications.	GESSA, Ana; JIMÉNEZ, Amor; SANCHA, Pilar.	2020	original research	n/a	online reviews and monitoring number of application downloads	the purpose of the paper is to understand the extent to which certified and non-certified mHealth apps influence user experience	The model showed a statistically significant partial association between "certification" and "online user reviews" (Z = 2.174; sig = 0.042)] and between "download" and "online user reviews" (Z = 2.387; sig = 0.017) However, quality certification labels as a guarantee of the quality of apps do not have a modulating effect on the online reviews and downloads relationship.
20	Innovations to improve access to musculoskeletal care.	CHEHADE, Mellick J., et al.	2020	original research	n/a	Literature review	to examine innovation, its management and the strategic directions required to improve musculoskeletal healthcare at macro (policy), meso (service delivery) and micro (clinical practice) levels.<br><br>to provide emerging evidence for how innovation can support systems' strengthening and build capacity to support improved access to the 'right' musculoskeletal care.	recommended systematic steps to establish required leadership, collaboration, research, networking, dissemination, implementation and evaluation of future innovations in musculoskeletal health and care.<br>The key steps required will not be realised without strong leadership supported by policy and funding and without the consumers

								strongly positioned and engaged across systems. We propose a number of recommendations for future innovation to support improved global musculoskeletal health and care.
21	Digital transformation in healthcare–architectures of present and future information technologies.	GOPAL, Gayatri, et al.	2019	review	n/a	Literature review	How can healthcare providers all over the world improve patient outcomes while containing costs with digital transformation.	Although many healthcare organizations have initiated digital transformation projects, very few have reached digital maturity.<br>It is key to build flexibility and insight into business processes through digital transformation, to rapidly respond to change, pivot towards the right outcomes, and ensure sustainable competitive advantage. This can only be achieved through digital transformation with intelligent, integration-ready applications and platforms that help to manage patients, networks, employees, and core processes.
22	A mobile phone-based program to promote healthy behaviors among adults with prediabetes who declined participation in free diabetes prevention programs: mixed-methods pilot randomized controlled trial	GRIAUZDE, Dina, et al.	2019	original research	n/a	parallel, 3-arm, mixed-methods pilot randomized controlled trial<br>participants were randomized to (1) a group that received information about prediabetes and strategies to prevent T2DM (control); (2) a group that received a mHealth app that aims to increase autonomous motivation among users (app-only); or (3) a group that received the app plus a physical activity tracker and wireless-enabled digital scale for self-monitoring (app-plus).	This study aims to examine the feasibility and acceptability of a mobile health (mHealth) intervention designed to increase autonomous motivation and healthy behaviors among adults with prediabetes who previously declined participation free DPPs.<br>In addition, the study aims to examine changes in autonomous motivation among adults offered 2 versions of the mHealth program compared with an information-only control group.	Overall, 28% (69/244) of eligible individuals were randomized; of these, 80% (55/69) completed the 12-week survey. Retention rates were significantly higher among app-plus participants than participants in the other 2 study arms combined (*P*=.004, χ^2). No significant differences were observed in adherence rates between app-only and app-plus participants (43 days vs 37 days; *P*=.34). Among all participants, mean autonomous motivation measures were relatively high at baseline (6.0 of 7.0 scale), with no statistically significant within- or between-group differences in follow-up scores. In qualitative interviews (n=15), participants identified reasons that they enjoyed using the app (eg, encouraged self-reflection), reasons that they did not enjoy using the app (eg, did not consider personal circumstances), and strategies to improve the intervention (eg, increased interpersonal contact).

23	Recommendations on the utilization of telemedicine in cardiology.	GRUSKA, Michael, et al.	2020	review	Austria	Literature review	aims to provoke a critical discussion of the digital change in cardiology and to make recommendations for the implementation of those telemedical processes that have been shown to exert positive effects on a wide variety of medical and economic parameters.	Telemedicine is intended to enable high-quality and cost-efficient care for an increasing number of patients, whose care poses one of the greatest challenges to our healthcare system. Not least of all, telemedicine should make a decisive contribution to improving the quality of life of this segment of the population by favorably influencing mortality, morbidity and hospitalization as well as the patient's contribution to treatment.
24	Telepsychiatry in the Arab World: A Viewpoint Before and During COVID-19.	EL HAYEK, Samer, et al.	2020	original research	n/a	Literature review and semi-structured guide with psychiatrists from different Arab nations	to describe the prior state and the changes that the COVID-19 outbreak brought to telepsychiatry in a selected group of Arab countries of the Middle East and North Africa (MENA) region	Before the pandemic, digital mental health services were provided in several Arab countries, mainly through hotlines and messaging services. The COVID-19 pandemic has marked a major shift in digital psychiatric services in the Arab MENA world, through the transformation of many clinics and some hospitals into digital mental health systems. Many non-governmental organizations also started remote initiatives for psychological support and psychiatric counseling. Three main barriers of patient-related, healthcare-related, and system-related hurdles of using telepsychiatry emanated from the analysis.
25	The digital transformation of the healthcare industry: exploring the rise of emerging platform ecosystems and their influence on the role of patients.	HERMES, Sebastian, et al.	2020	original research	n/a	to investigate the digital transformation of the healthcare industry by analyzing 1830 healthcare organizations found on Crunchbase.	to incorporated an interorganizational perspective of the digital transformation of healthcare	The results indicate 8 new roles within healthcare, namely: information platforms, data collection technology, market intermediaries, services for remote and on-demand healthcare, augmented and virtual reality provider, blockchain-based PHR, cloud service provider, and intelligent data analysis for healthcare provider. Results further illustrate how these roles transform value proposition, value capture, and value delivery in the healthcare industry.

26	Artificial intelligence: power for civilisation– and for better healthcare.	HORGAN, Denis, et al.	2019	review	n/a	Literature review	to set out how AI can bring new precision to care, with benefits for patients and for society as a whole<br><br>and<br><br>to set out the conditions for realizing the potential.	The conditions for realizing the potential of AI are: ensuring adequate access to data, an appropriate regulatory environment, action to sustain innovation in research institutes and industry big and small, promotion of take-up of innovation by the healthcare establishment, and resolution of a range of vital legal and ethical questions centred on safeguarding patients and their rights<br>Europe to fulfil the conditions for success, it will have to find a new spirit of cooperation that can overcome the handicaps of the continent's fragmented technical and legal landscape.
27	Therapist-supported online interventions for children and young people with tic disorders: Lessons learned from a randomized controlled trial and considerations for future practice.	CHAMBERLAIN, Liam R., et al.	2020	original research	n/a	experiences and reflections of 4 therapists and their 2 supervisors in delivering an online, therapist-supported intervention in a randomized controlled trial for children and young people with tic disorders	to investigate implementation of internet-based cognitive behavioral therapy (iCBT) in practice and what aspects of the therapist role support success.	this paper highlights important points for consideration when delivering remote iCBTs, including adequate therapist training, clinical supervision, flexibility, and organization.<br>Further this paper suggests how therapist-guided iCBTs could fit into pre-existing services.
28	Artificial intelligence in healthcare: An essential guide for health leaders.	CHEN, Mei; DECARY, Michel.	2020	review	n/a	Literature review	to describe the state of AI technologies and the potential of such technologies for transforming healthcare<br>and<br>to provide a guide to understand the fundamentals of AI technologies (ie, machine learning, natural language processing, and AI voice assistants) as well as their proper use in healthcare.<br>to provide practical recommendations to help decision-makers develop an AI strategy that can support their digital healthcare transformation.	The development and implementation of AI in healthcare is complex and costly, so health organizations need to make smart decisions and develop strategic plans that enable them to bring real value to their organizations.<br>For example: Considering both short-term and long-term goals of your organization, Establishing the leadership, team, culture, and collaboration for successful implementation, Selecting the right AI platform, tools, and approaches for implementing your AI strategy, Forming a good data strategy to derive patient insights, Determining the context and protocols for the safe use of AI technology, Developing nationwide AI-powered digital healthcare ecosystems ...

29	Digital innovation in healthcare: a device with a method for monitoring, managing and preventing the risk of chronic polypathological patients	IMPROTA, Giovanni, et al.	2020	Perspective, opinion, and commentary	USA	revies, synthesis, process maps, examples	to present a novel patented device for automatic processing of clinical data of chronic poly-pathological patients	The invention consists of a reconfigurable equipment that allows the assessment of clinical risk severity indexes that can be customized for polypathological patients and which acts both as a decision support system for specialist doctors in the diagnosis and treatment phases, and as a monitoring system in the clinical environment
30	Evaluating healthcare practitioners' views on store-and-forward teledermoscopy services for the diagnosis of skin cancer.	JANDA, Monika, et al.	2019	original research	n/a	online survey	to evaluate healthcare practitioners' views on and satisfaction with (i) digital image acquisition and storage and (ii) store-and-forward teledermoscopy services for the diagnosis of skin cancer in their clinical practice	Thirty-four healthcare practitioners (58%) had previously used a mobile dermatoscope within their practice. Participants most appreciated its use in their practice for lesion monitoring (59%) and record keeping (39%). Challenges reported were the increased time to support the additional workload (45%), technical issues (33%) and cost of equipment (27%). Practitioners were unsure (36%) or did not advocate teledermoscopy for direct-to-consumer use (41%). Only 23% supported the use of direct-to-consumer teledermoscopy.
31	Artificial intelligence biosensors: Challenges and prospects.	JIN, Xiaofeng, et al.	2020	review	n/a	Literature review	to summarize the most advanced progress made in the key phases for future wearable and implantable technology from biosensing, wearable biosensing to AI-biosensing<br>and<br>to discuss the challenges and opportunities of AI-biosensors moving forward toward future medicine devices	Much efforts should be devoted to developing flexible electronic materials for integrating chip technology, IoT, big data and AI to realize self-learning, self-assembly and self-adaptation of AI-biosensor systems.<br>Emphasis is placed on the related fields of flexible bioelectronic materials and integration, wireless communication, machine learning and smartphone-based platforms for the development of AI-biosensors.
32	Digital health: a new dimension in rheumatology patient care.	KATARIA, Suchitra; RAVINDRAN, Vinod.	2018	review	n/a	Literature review	to update on the recent advances in the field of digital health and highlight unique features of these technologies which would help in routine care	Digital applications are not the panacea to all the shortcomings in patient care as the human touch can never be replaced by any means. Technology is evolving constantly and with that newer approaches in digital health will keep emerging. The patients are now more informed due to the

								accessibility of information and are taking self-responsibility in managing the disease better. More observational studies, randomized controlled trials, systematic review and meta-analysis of digital health efficacy in rheumatology health care are the need of the hour.
33	Impact of mobile health and medical applications on clinical practice in gastroenterology.	KERNEBECK, Sven, et al.	2020	review	n/a	Literature review	to provide an overview of the current status of MHA and MA use in the field of gastroenterology, describe the future perspectives in this field and point out some of the challenges that need to be addressed.	Digital interventions, such as MHAs and MAs, offer potential for diagnostic and treatment advances in the field of gastroenterology and the management of chronic diseases in general. In particular, patients with chronic diseases and health care professionals will benefit from these interventions in many different ways. Sufficient proof of benefit, however, depends on high-quality evaluation, which must be based on the standards of evidence-based medicine. This issue is complicated for digital interventions for many reasons, and to date, the specific standards for development and evaluation are generally missing. In this context, it should be clearly emphasized that frameworks of standardization, at least in many parts, can harmonize the research in the field of digital interventions. Continuous work on standardization with a clear focus on the rules of evidence-based medicine would lead to a better understanding and interpretation of the actual evidence.
34	Managing as Designing: Transforming Digital Healthcare Interoperability	KOBUSINGE, Grace	2020	conference paper/original research	Sweden	interviews, synthesis framework	to investigate a managing as designing (MaD) approach taken by a successful HIS interoperability initiative in Sweden	This study highlights the importance of analyzing the 'institutional context of integration' in order to align the context to an appropriate interoperability principle. The study also demonstrates the importance of analyzing the existing HIS and other pressing internal and external contextual factors including policy and required resources.

								Consequently, through constant analysis of existing HIS, factors concerning system diversity/heterogeneity, automaticity, homogeneity have to be scrutinized, as well as assessing system interoperability capabilities. Subsequently, the context analysis exercise leads to a set of interoperability design requirements that can guide the HIS implementation process. Thus, through continuous institutional context analysis, decisions to unify existing systems, design an intersection between them, discard the systems or leave systems as they are might be reached.
35	Without data, you're just another person with an opinion	KOLASA, Katarzyna, et al.	2020	review	n/a	Literature review	to explore how new streams of data will contribute to further the transformation of the healthcare system across different jurisdictions. "How the growing amount of data will change the way we assess the value of healthcare technologies, ensuring a more holistic approach in the decision-making process, or in the search for waste reduction in healthcare?"	In sum, we acknowledge that the role of Big Data in the transformation of healthcare in an era of limited resources will continue to grow. Still, however, the current implementation is mainly ad hoc and generally on a local level. New frameworks where the patient will be genuinely empowered to handle his/her health and data will be needed. There is still underutilisation of the multiple data sources because of the current unstructured approach. This is especially true for complex, noisy longitudinal, and voluminous data. Therefore, clinicians should work with data analysts to establish a clinically meaningful reporting format. Access to Big Data is not only important at the national level but also at the international level. The collaboration in the field of diagnostics and treatment can increase the knowledge volume. Such a possibility will certainly enrich the research potential, especially in areas where

								knowledge is limited. Some authors suggest that value and variability truly define Big Data. This provides hope in anticipation of the future. There is no problem with innovation in the field of data analytics in healthcare but there is a problem with its adaptation.
36	Wearable health devices and personal area networks: can they improve outcomes in haemodialysis patients?	KOOMAN, Jeroen P., et al.	2020	review	n/a	Literature review	to discuss potentially relevant developments for haemodialysis patients that may help dialysis caregivers of the future to improve outcomes in haemodialysis patients	It is very likely that, also driven by rapid technical and societal changes, wearable health devices WHD will enter clinical practice in the near future, including for dialysis patients. Whereas healthcare professionals may initiate some of these developments, patient preferences and a technology push from device manufacturers will also likely play a major role. Whereas the introduction of WHD, certainly when accompanied by adequate feedback and educational modules, could play a major role in the 4P healthcare of the future, a major challenge is to balance societal expectation with the workload and liability of healthcare professionals.
37	The new European interoperability framework as a facilitator of digital transformation for citizen empowerment.	KOUROUBALI, Angelina; KATEHAKIS, Dimitrios G.	2019	review	n/a	Literature review	to propose a new methodological approach for overcoming barriers to the adoption of digital tools for citizen empowerment and other forms of patient-centered eHealth and to offer a policy viewpoint on how the new European Interoperability Framework (EIF) may benefit the implementation of eHealth systems for the management of personal health information for citizens	An interoperability framework is necessary to provide the conditions so that digital tools are adopted and used in a trustworthy manner. It is evident that no solution will fit all circumstances. However, the new EIF, when adapted for personally managed health data, provides a useful and relevant framework to facilitate implementation and adoption of personal health record systems within a coordinated care environment. Practical implications of this work relate to the need of multi-disciplinary cooperation and European level compatibility and sustainability of the underlying infrastruc-

								tures required to support reliable and secure access to and sharing of medical data, as well as the readiness to address continuously evolving functional and non-functional requirements for regional, national, and cross-border settings.
38	Digital biomarkers for Alzheimer's disease: the mobile/wearable devices opportunity	KOURTIS, Lampros C., et al.	2019	review	n/a	Literature review	to review existing early clinical manifestations of AD and a path to the respective sensor and mobile/wearable device usage to acquire domain-centric data towards objective, high frequency and passive digital phenotyping; to define list of sensors and their respective domains and metrics	While the debate on the preferred course of action is still on and it involves among others, regulatory, ethical, legal, data privacy and clinical considerations, some options involve: -Notifying the user that there is something out of the normal with his/her longitudinal rate of progression of neurological health, so he/she can seek further clinical assessment. -Providing longitudinal disease-related digital biomarkers to a healthcare practitioner, to allow for objective and continuous clinical evaluation of a user.
39	Digital triage: Novel strategies for population health management in response to the COVID-19 pandemic.	LAI, Lucinda, et al.	2020	original research	USA	Case Study - description	to build an online screening and triage tool to distinguish between patients who were overall well from patients requiring more immediate attention	There was an increased utilization during the period of March–April 2020 reflecting the overall success of the chatbot implemented. Patients needing additional assessment were directed to the hotline clinician to determine where to direct the patient: drive-through testing sites for those who met testing criteria but were well or mildly ill and free of pertinent comorbities, respiratory illness clinics for those with moderate comorbidities and/or symptoms requiring in-person evaluation, telemedicine consultation with a physician, or proceed immediately to the nearest ED for the severely ill. AI Triage tool decreased the burden of work and serves as an exemplar for the integration of automated technologies into human work flows.

40	A Showcase of Medical, Therapeutic and Pastime Uses of Virtual Reality (VR) and How (VR) Is Impacting the Dementia Sector	LEE, Suzanne	2019	review	n/a	literature review	to summarize the virtual reality benefits and advantages for dementia	Virtual reality has many positive effects for the diagnosis of dementia, there are also many questions that need to be addressed (VR content, policies, resources, problem solvers, developers etc.)
41	Anesthesia in the modern world of apps and technology: Implications and impact on wellness	LO, Calvin, et al.	2021	Special Interest Article	Canada	literature review	1. to examine examples of novel technology and the impact of these digital interventions on the anesthesiologist's well-being<br>2. to review popular personalized technology aimed at improving wellness and the impact on well-being examined.<br>3. to introduce how to improve technology adoption, which, when appropriately applied, may minimize the negative impacts of technology on anesthesiologists' well-being.	Recognizing factors that affect technology acceptance can provide a starting point for technology implementation. Incorporating quantitative, serial assessments of well-being as part of technology implementation are proposed as a future direction for examining the wellness impact of technology on anesthesiologists.
42	Flexible hybrid electronics for digital healthcare	MA, Yinji, et al.	2020	original research	n/a	synthesis	1.to introduce structural design for the preparation of flexible hybrid electronics,<br>2.to give a brief chronology of these advances,<br>3.to describe the biomedical applications in bioelectrical monitoring and stimulation, optical monitoring and treatment, acoustic imitation and monitoring, bionic touch, and body-fluid testing are described.	Collectively, the advances in materials and mechanics of<br>flexible hybrid electronics provide potential for applications in tissue engineering and biomedical fields.<br>There ase several challenges:<br>1.designing and integrating more units/functions in a space-limited device<br>2. energy source - (from the needs of flexible devices in terms of reliability and power level)<br>3. large-scale manufacturing technology.
43	Medicine of the future: digital technologies in healthcare	MAMYRBEKOVA, Saltanat, et al.	2020	conference proceeding	n/a	SWOT analysis review synthesis	to discusses the role of innovative digital technologies in medicine to improve its competitiveness and efficiency of organization and management in health care, as well as improving the quality of treatment	positive trends in the implementation of it in practical health care were identified:<br>1) improved adherence to treatment, in particular according to the percentage of patients who actively use home

								self-monitoring methods increases to 90%.<br>2) decrease in the frequency of hospitalizations of patients improving the quality of life, psychological and social standing of the patient;<br>3) reduction of mortality among patients with cardiovascular diseases by 20-25% compared to the routine technology of medical care organization, i.e. without the use of it systems;<br>4) increasing patient satisfaction with medical services and improving the quality of life;<br>5) increasing patient awareness of their disease;<br>6) improving the quality of service, timely correction of drug therapy, high efficiency of drug treatment;<br>7) improving the economic efficiency of medical care
44	Use of the HoloLens2 Mixed Reality Headset for Protecting Health Care Workers During the COVID-19 Pandemic: Prospective, Observational Evaluation	MARTIN, Guy, et al.	2020	original research	UK	Pilot project:<br>A prospective, observational, nested cohort evaluation of the device was undertaken across 3 distinct clinical clusters in a teaching hospital.<br>High-level aggregate outcome data pertaining to staff exposure to high-risk COVID-19 environments and PPE use were collected.<br>Assessments of feasibility and acceptability were undertaken via user experience questionnaires with Likert and free-text	to deploy the HoloLens2 MR device to support the delivery of remote care in COVID-19 hospital environments	Deploying such technologies at pace requires context-specific information security, infection control, user experience, and workflow integration to be addressed at the outset and led by clinical end-users. The deployment of new telemedicine technology must be supported with objective evidence for its safety and effectiveness to ensure maximum impact.<br><br>The deployment of the HoloLens2 led to a 51.5% reduction in time exposed to harm for staff looking after COVID-19 patients (3.32 vs 1.63 hours/day/staff member; P=.002), and an 83.1% reduction in the amount of PPE used (178 vs 30 items/round/day; P=.02). This represents 222.98 hours of reduced staff exposure to COVID-19, and 3100 fewer PPE items used

						responses.		each week across the three clusters evaluated. The majority of staff using the device agreed it was easy to set up and comfortable to wear, improved the quality of care and decision making, and led to better teamwork and communication. In total, 89.3% (25/28) of users felt that their clinical team was safer when using the HoloLens2.
45	The technological imperative for value-based health care	MEINERT, Edward, et al.	2018	review	n/a	synthesis	to help understand the tools and factors associated with the use of technology for the delivery of a value-based health-care system	1. The complex nature of health-care systems creates a challenge for adoption of a value-based approach.<br>2. Technology is an enabler which can provide detailed monitoring of outcomes and costs, while facilitating change in processes and delivery of care.<br>3. Opportunities for technology to facilitate a value-based health-care system need to take into account the following trends: creating a value-enabling information technology platform, interoperability, user experience, patient empowerment, disintermediation of care and real-time analytics.
46	The internet of things in health care in oxford: protocol for proof-of-concept projects	MEINERT, Edward, et al.	2018	protocol	UK	This study describes the PoC projects that will be created to explore cost-effectiveness, clinical efficacy, and user adoption of Internet of Medical Things systems<br>-	is to provide:<br>1. an overview of the current state of Internet of Things (IoT) and key implementation considerations,<br>2. key use cases demonstrating technology capabilities,<br>3. an overview of the landscape for health care IoT use in Oxford,<br>4. recommendations for promoting the IoT via collaborations between higher education institutions and industry proof-of-concept (PoC) projects.	The study started in March 2018, and results are expected by the end of 2019.<br>The aims of this project is as follows:<br>1. To enable EHR-integrated smart device interoperability,<br>2. To demonstrate accessibility to various devices across the same disease state, and<br>3. To compare use of personal devices with controlled medical devices and evaluate adoption and clinical data accuracy.

47	Inclusive innovation in telehealth	NOEL, Kimberly; ELLISON, Brooke	2020	Perspective, opinion, and commentary	n/a	n/a	n/a	n/a
48	Design and development of referrals automation, a SMART on FHIR solution to improve patient access to specialty care.	ODISHO, Anobel Y., et al.	2020	original research	USA	description of implementation process and effects of innovation	to describe the design process and architecture for a software application that has been developed and deployed to optimize the referrals intake process by automating the processing and digitization of incoming specialty referral faxes, extracting key data elements and integrating them into the electronic health record (EHR), and organizing referrals.	The human-centered design process described in this manuscript identified and quantified steps related to the referral process that delay patient access to specialty care. Tasks such as the manual entry of information from an incoming fax emerged as immensely time-consuming, tedious, error-prone, and well-suited to automation. From a methodology and technological standpoint, this application yields several advantages. First, the application aims to improve the turnaround time from referral receipt to a scheduled appointment by automating key steps in the patient referral workflow. Second, the referrals user interface promotes greater organization and accountability to complete scheduling of patient referrals. Third, the application was built using the SMART on FHIR standard, allowing for adaptability and future use by other healthcare organizations. This application has been deployed widely at our large tertiary and quaternary care institution, minimizing the manual entry of faxed data and streamlining the intake process for incoming referrals.
49	Hearing Implants in the Era of Digitization.	OLZE, Heidi, et al.	2019	review	n/a	Literature review	In this study, current developments from the perspective of clinical practice providing cochlear implants (CI) are presented and discussed.	The study illustrates a wide range of digital applications that can be used in all phases of CI care, from patient information through hearing screening and preoperative assessment options to lifelong follow-up care and clinical research.

50	Emergence of new disease: how can artificial intelligence help?	PARK, Yurim, et al.	2020	review	n/a	Literature review	to review the recent applications of AI across disease prediction and drug development in relation to the COVID-19 pandemic	Applications of AI in relation to the COVID-19:<br>1) to predict viral mutation before a new strain has even emerged.<br>2) to mine existing data for insights on how to treat the disease.<br>3) to understand viral structures can accelerate the process of drug development<br>Putting aside practical applications, the ethical and societal implications of these new technologies must also be considered, and will require systematic examination, (eg, issues around security, privacy, and confidentiality)
51	Artificial Intelligence in Health Care: Current Applications and Issues	PARK, Chan-Woo, et al.	2020	review	n/a	literature review/short	to introduce the current research and application status of AI technology in health care and discuss the issues that need to be resolved	current issues in healthcare were defined:<br>- Issues of utilizing health care data<br>- Regulatory affairs and policies for new devices<br>- Safety and liability issues<br>- Balanced application with existing health care systems
52	Artificial intelligence in medical imaging: threat or opportunity? Radiologists again at the forefront of innovation in medicine.	PESAPANE, Filippo; CODARI, Marina; SARDANELLI, Francesco.	2018	review	n/a	Literature review	to provide basic definitions of terms such as “machine/deep learning” and analyse the integration of AI into radiology<br>and<br>to provide an overview of the balance between AI threats and opportunities for radiologists	AI will surely impact radiology, and more quickly than other medical fields.<br>With an irreversible increase in the amount of data and the possibility to use AI to identify findings either detectable or not by the human eye, radiology is now moving from a subjective perceptual skill to a more objective science. Radiologists, who were on the forefront of the digital era in medicine, can guide the introduction of AI into healthcare. Yet, they will not be replaced because radiology includes communication of diagnosis, consideration of patient’s values and preferences, medical judgment, quality assurance, education, policy-making, and interventional procedures. The higher efficiency provided by AI will allow radiologists to perform more value-added tasks, becoming more visible to patients and

								playing a vital role in multidisciplinary clinical teams.
53	Virtual telemedicine visits in pediatric home parenteral nutrition patients: a quality improvement initiative.	RAPHAEL, Bram P., et al.	2019	original research	n/a	collection of data on pediatric patients managed at a single HPN program who participated in postdischarge telemedicine visits	to evaluate effectiveness of telemedicine in pediatric home parenteral nutrition (HPN)	Telemedicine visits identified opportunities for improvement for families newly discharged on HPN. In a small cohort of patients who experienced telemedicine visits, we found lower central line-associated bloodstream infections (CLABSI) rates alongside higher readmission rates compared with a historical comparison group. Further studies are needed to optimize telemedicine in delivering care to this high-risk population.<br>Compared to historical comparison group, the telemedicine group experienced CLABSI rates of 1.0 versus 2.7 per 1,000 line days and readmission rates of 38% versus 17% ($p = 0.03$, 0.02, respectively).
54	IDF Europe's position on mobile applications in diabetes.	ROSE, Kyle Jacques, et al.	2019	review	n/a	Literature review	to think about Mobile Applications in Diabetes, examining Diabetes and new technology through psychology, motivation and behavioral change in diabetes management; the healthcare professional perspective; potential roles of diabetes-related Apps, pointing to existing evidence and important ethical issues; and<br>to offer recommendations on four levels: individual, healthcare professional, political and App developers.	Thousands of Apps are available, targeting different audiences and strategies to prevent and manage diabetes.<br>There is tremendous potential in the ability of these Apps to make an impact on the lives of people with diabetes in all corners of our Region, overcoming obstacles to access which may exist.<br>Patients and providers must recognize the characteristics of these products and services to capitalize on the advantages while avoiding harmful deficiencies.<br>Recommendations have been defined for individual, healthcare professional, political and App developers.
55	Going digital: a checklist in preparing for hospital-wide electronic medical record implementation and digital transformation.	SCOTT, Ian A.; SULLIVAN, Clair; STAIB, Andrew.	2019	original research	n/a	Literature review, workshop with multidisciplinary group, a draft document based on literature and workshop proceedings	to develop a checklist that clearly and comprehensively defines the steps that best prepare hospitals for EMR implementation and digital transformation.	The final checklist comprised 19 questions, 13 related to EMR implementation and six to digital transformation. Questions related to the former included organisational considerations (leadership, governance, change leaders, implementa-

								tion plan), technical considerations (vendor choice, information technology and project management teams, system and hardware alignment with clinician workflows, interoperability with legacy systems) and training (user training, post-go-live contingency plans, roll-out sequence, staff support at point of care). Questions related to digital transformation included cultural considerations (clinically focused vision statement and communication strategy, readiness for change surveys), management of digital disruption syndromes and plans for further improvement in patient care (post-go-live optimisation of digital system, quality and benefit evaluation, ongoing digital innovation).
56	Are innovation and new technologies in precision medicine paving a new era in patients centric care?	SEYHAN, Attila A.; CARINI, Claudio.	2019	review	n/a	Literature review	to describe impact of digital innovations on drug development	The drug development is a challenging long process with many obstacles on the way. Though several strategies have been proposed to tackle this issue, there is a general consensus that a better use of BMs, omics data, AI and machine learning will accelerate the implementation of a new medical practice that will depart from the widely spread concept “one drug fits all”.
57	Artificial intelligence in diagnostic imaging: status quo, challenges, and future opportunities.	SHARMA, Puneet, et al.	2020	review	n/a	Literature review	to discuss the current and future impact of artificial intelligence (AI) technologies on diagnostic imaging, with a focus on cardio-thoracic applications.	The processing of imaging data is described at 4 levels of increasing complexity and wider implications.<br>1) At the examination level, AI aims at improving, simplifying, and standardizing image acquisition and processing.<br>2) + 3) At the reading and reporting levels, AI focuses on automatic detection and characterization of features and on automatic measurements in the images.<br>4) At the prediction and prescription levels, AI focuses on risk prediction and stratification, as opposed to merely detecting, measuring, and quantifying images.

58	Implementation of a Centralized Telepsychiatry Consult Service in a Multi-Hospital Metropolitan Health Care System: Challenges and Opportunities.	SHAYEVITZ, Christina, et al.	2021	original research	n/a	The pilot study - resulted in 557 completed telepsychiatry consults over the course of 13 months from 2018 to 2019	1.to describe the implementation, interprofessional workflow, process of triage, and provider satisfaction 2.to demonstrate feasibility of a hub and spoke model for provision of inpatient consult telepsychiatry service from an academic medical center to 2 affiliated regional hospital sites, to reduce patient wait time, and to develop best practice guidelines for telepsychiatry consultations to the acutely medically ill.	This study demonstrated the feasibility of a centralized telepsychiatry hub to improve delivery of psychiatry consultation within a multihospital system with an overall reduction in patient wait time. . A range of psychiatric conditions commonly encountered by consultation-liaison services were diagnosed and treated through the teleconferencing modality. The most common barriers to successful use of telepsychiatry were defined for the 20% of consult requests that were retriaged to face-to-face evaluation. The average patient wait time from consult request to initial consultation was reduced from >24 hours to 92 minutes.
59	A Theoretical Framework and Conceptual Design for Engaging Children in Therapy at Home—The Design of a Wearable Breathing Trainer	SIERING, Lara, et al.	2019	original research	Germany	1. The Design for Child Engagement (DCE) Framework 2. Evaluation by experts and by the target group	1.to perform the design of a wearable breathing trainer for children with asthma and dysfunctional breathing 2. to provide knowledge about the implementation of theory on engagement and motivation in design	The framework proved useful to inform the design process to design a wearable device in mainly two ways. First, it provided inspiration as a starting point for design, second, it provided a solid ground to make design decisions. Some important decisions made based on the DCE Framework during the design process were later confirmed to match the target groups needs in the evaluation. For example, the decision to use light in the form of an LED matrix with a focus on the quantitative aspect as feedback on performance was evaluated as markedly explicit and understandable. Another important design decision that was made based on the framework was to combine the vest with a mobile application. With using the data on performance from the vest to increase the challenge, it is guaranteed that the child will stay engaged during the therapy
60	Precompetitive Consensus Building to Facilitate the Use of Digital Health Technologies to Support Parkinson Disease Drug	STEPHENSON, Diane, et al.	2020	review	n/a	article examples, synthesis	to highlight how collaborations can address gaps in the application of digital health technologies (DHT) with Parkinson disease as the key focus;	The Critical Path for Parkinson's Consortium of the Critical Path Institute is highlighted as a case example where stakeholders collectively engaged regulatory agencies on the effective use of DHT in PD

	Development through Regulatory Science						to analyse collaborative initiatives that supports the implementation of DHT	clinical trials. Global regulatory agencies, including the US Food and Drug Administration and the European Medicines Agency, are encouraging the efficiencies of data-driven engagements through multi-stakeholder consortia
61	The evolution of mHealth solutions for heart failure management	TRIPOLITI, Evanthia E., et al.	2018	review	n/a	literature review, comparison, synthesis	to provide an overview of (i) the current practice in the management of heart failure, (ii) the available mHealth solutions, either in the form of the commercial applications, research projects, or related studies, and (iii) the several challenges related to the patient and healthcare professionals' acceptance, the payer and provider perspective, and the regulatory constraints.	Taxonomy of mHealth-based HF interventions is performed Comparison of mHealth research projects related to heart failure (HF) is performed MARS – Mobile Application Rating Scale score is introduced
62	In-Hospital telehealth supports care for neonatal patients in strict isolation	UMOREN, Rachel A., et al.	2020	original research	USA	In February 2020, within the setting of a children's hospital intensive care unit with patients, an InTouch Vici telemedicine cart was used to establish a secure, encrypted connection with a Microsoft Surface Pro tablet device in the ICU. A Littmann 3200 digital bluetooth stethoscope was used with the cart.	to determine the feasibility of "in-hospital" inpatient telemedicine within a children's referral hospital to facilitate inpatient care activities such as interprofessional rounding and the provision of supportive services such as lactation consultations to pediatric patients in strict isolation. To test the feasibility of in-hospital video telemedicine, a dedicated telemedicine device was set up in the patient's room.	Telehealth supports patient care in isolation. Telehealth reduced health care provider exposures. Telehealth conserves personal protective equipment.
63	Analysis of factors affecting IoT-based smart hospital design	USLU, Banu Çalış; OKAY, Ertuğ; DURSUN, Erkan	2020	original research	n/a	review, synthesis, Cause and effect diagram, process maps	1.to point out the optimization factors, challenges, available technologies, and opportunities, as well as the system architecture that come about by employing IoT technology in smart hospital environments. 2.to design technical infrastructure	The technical infrastructure is divided into five layers and the system infrastructure, constraints, and methods needed in each layer are specified, which also includes the smart hospital's dimensions and extent of

								intelligent computing and real-time big data analytic. The deficiencies that may arise in each layer for the smart hospital design model and the factors that should be taken into account to eliminate them are explained. It is expected to provide a road map to managers, system developers, and researchers interested in optimization of the design of the smart hospital system.
64	Big data requirements for artificial intelligence	WANG, Sophia Y., et al.	2020	review	n/a	literature review	To summarize how big data and artificial intelligence technologies have evolved, their current state, and next steps to enable future generations of artificial intelligence for ophthalmology.	Future requirements for big data and artificial intelligence include fostering reproducible science, continuing open innovation, and supporting the clinical use of artificial intelligence by promoting standards for data labels, data sharing, artificial intelligence model architecture sharing, and accessible code and APIs.
65	Utilising digital health to improve medication-related quality of care for hypertensive patients: An integrative literature review	WECHKUNANUKUL, Kannikar; PARAJULI, Daya Ram; HAMIDUZZAMAN, Mohammad	2020	review	n/a	An integrative literature review was undertaken in October 2019 using the Medline, Cumulative Index of Nursing and Allied Health Literature, and Scopus databases for publications in English with no date limit	To summarise the outcomes from a range of research which investigated the use of DHI to improve the medication-related quality of care (MRQOC) for hypertensive patients	In total, 18433 participants were included in this review from 28 studies meeting the eligibility criteria. There were 19 DHI identified within eight countries: Australia, Canada, India, South Korea, Lebanon, Pakistan, the United Kingdom, and the United States of America. The DHI were provided as community-based, clinical-based and home-based program through mobile phone, mobile health system, short message service, and telehealth, digital medicine, and online healthcare (web-based). The mean age of participants was 59 ranging from 42 to 81 years with an average mean systolic blood pressure of 143.3 mmHg at baseline, ranging from 129.0 mmHg to 159.0 mmHg. The proportion of male participants ranged from 13.9% to 92.0%. Eighteen interventions showed evidence of reduction in blood pressure and improvement of self-

								management in relation to medication adherence and blood pressure control. The reduction of systolic blood pressure ranged between 1.9 mmHg and 26.0 mmHg, with a mean of 10.8 mmHg. The digital health was found positively associated with the MRQOC for hypertensive patients such as improvement in medication adherence and medication management; better blood pressure control; maintaining follow-ups appointment and self-management; increasing access to healthcare particularly among patients living in rural area; and reducing adverse events. However, some interventions found no significant effect on hypertensive care. The follow up duration varied between 2 mo and 18 mo with an average attrition rate of 10.1%, ranging from 0.0% to 17.4%
66	Connected health technology for cardiovascular disease prevention and management	WONGVIBULSIN, Shannon, et al.	2019	review	n/a	review, synthesis	to review the current evidence supporting the use of connected health technologies for the prevention and management of cardiovascular disease in an effort to highlight gaps and future opportunities for innovation.	Cardiovascular disease prevention and management continue to be key focus areas for clinicians and researchers in the connected health space. Exciting progress has been made though studies continue to suffer from small sample size and limited follow-up. Efforts that combine home patient monitoring, engagement, and personalized feedback are the most promising. Ultimately, combining patient-level ambulatory sensor data, electronic health records, and genomics using machine learning analytics will bring precision medicine closer to reality.
67	Advanced data analytics for clinical research part I: what are the tools?	ZHOU, Nicolas, et al.	2020	review	n/a	review, synthesis	to explore the tools for Cardiothoracic surgeons pursposes: Advanced data capture and categorization tools such as natural language processing and optical	With recent advances in technology and Big Data infrastructure, the future of healthcare is currently undergoing dynamic transformation. Big Data has the promise to provide more clear insight into our patients' disease processes through more avenues of data and more powerful

	character recognition are being developed for clinical and research use, while Internet of Things in the form of wearable technology serves as an additional source of data usable for research.<br>As cardiothoracic surgeons seek ways to innovate, novel approaches to data acquisition and analysis enable a more rigorous level of investigatory efforts.	computing. Many hospitals in the United States and around the world already have or will eventually have Big Data infrastructure for their patient data. In understanding the Big Data infrastructure and advances in AI technology, researchers can begin to form new research questions and methods that address questions never before asked.<br>Tools such as natural language processing (NLP) and optical character recognition (OCR) are tools being utilized to facilitate research processes, while IoT and wearable technologies open the door for researchers to capture patient health outside of the hospital. With the rising cost of healthcare and demand for better outcomes, this industry is ripe for disruption. Learning to embrace the data transformation and leveraging our role as clinicians will serve to better patient outcomes.
